# Clinical and cost benefit of rapid molecular screening for carbapenem-resistant organisms: a seven-year analysis in a tertiary hospital

**DOI:** 10.1186/s13756-026-01750-7

**Published:** 2026-05-10

**Authors:** Riccardo Bollini, Davide F. Bavaro, Francesco De Fazio, Raffaella Renzulli, Cristina Scuderi, Alessandra Belati, Veronica Ciorba, Alessandra Calabrese, Benedetta Varisco, Filippo Medioli, Linda Bussini, Zian Asif, Sara Carloni, Giorgio Da Rin, Antonio Voza, Maurizio Cecconi, Elena Vanni, Erminia Casari, Michele Bartoletti, Valeria Cento

**Affiliations:** 1 Department of Public Health, Experimental and Forensic Medicine, University of Pavia, 27100 Pavia, Italy; 2 Humanitas University, Via Rita Levi Montalcini 4, 20072 Pieve Emanuele, Milan, Italy; 3 IRCCS Humanitas Research Hospital, Via Manzoni 56, 20089 Rozzano, Milan, Italy

**Keywords:** Rectal colonization, Carbapenemase, Enterobacteriaceae, Bloodstream infection

## Abstract

**Background:**

The endemic spread of carbapenem-resistant organisms (CROs) is often driven by inadequate prevention of patient-to-patient transmission. This study evaluates the impact of rapid molecular surveillance on in-hospital CRO spread, invasive infections, and costs.

**Methods:**

In this retrospective, single-center study (Jan 2017–Dec 2023), adult patients at IRCCS Humanitas Research Hospital underwent CRO simultaneous screening using PCR (Xpert™ Carba-R) and culture (Brilliance™ CRE Agar) on admission, with weekly follow-ups in ICUs and onco-hematology wards. Upon PCR result availability, patients were isolated/cohorted. Bloodstream infections (BSIs) were defined by positive blood cultures. Cox regression estimated the risk of incident BSIs. In parallel an economic analysis of the two methods was conducted to asses their impact on resources.

**Results:**

Among 21,525 samples, PCR and culture showed high concordance (Cohen’s Kappa: 0.909, 95% CI: 0.89–0.93). PCR excluded CRO colonization with 99.7% specificity (95% CI: 99.61–99.76) and a negative likelihood ratio of 0.02. Negative PCR results were available in a median of 2.6 h versus 50 h for culture (*p* < 0.001), reducing isolation time and adding ~ 10 bed-days annually in our two-bed setup. PCR sensitivity for culture-proven colonization was 97.7% (95% CI: 96.2–99.18). *K. pneumoniae* accounted for 88% of isolates, with blaKPC as the predominant gene, followed by blaVIM (32.1% culture-negative). CRO acquisition rates were low in ICUs (1.3/1000 patient-days) and onco-hematology wards (0.32/1000 person-days). CRO-BSIs occurred in 75 patients after a median of 12 days, with incidences of 2.35/1000 person-days in colonized versus 0.17/1000 in non-colonized patients. Cox regression identified acquired CRO colonization (aHR = 8.11, 95% CI: 2.38–27.61) and pre-existing blaNDM colonization (aHR = 6.23, 95% CI: 1.05–36.99) as independent predictors of CRO-BSI. The short turnaround times of molecular approach allows to free up beds, increasing the number of patient treatable each years.

**Conclusions:**

PCR screening is highly concordant with culture, while delivering faster results with positive impacts on isolation time, bed availability, and rapid identification of patients at high risk for CRO-BSIs. From an economic perspective, the molecular approach leads to an optimization of scarce and precious resources.

**Supplementary Information:**

The online version contains supplementary material available at 10.1186/s13756-026-01750-7.

## Background

The spread of carbapenem-resistant microorganisms (CRO) has been rapid on a global scale, where they cover top positions in the WHO top pathogen list. CROs indeed represents a significant (and escalating) challenge in nosocomial infections worldwide, due to transmissibility and high mortality rates associated with incident infections and the limited treatment options available [1, 2].

Asymptomatic CRO colonization in the gastrointestinal tract often precedes invasive infections. Studies have shown that patients colonized with CRO have a significantly higher risk of developing CRO infections compared to non-colonized [3, 4], and these patients constitute a relevant source of in-hospital transmission for multidrug-resistant pathogens.

Active surveillance to identify asymptomatic carriers of CRO is therefore one of the most critical (and recommended) components of infection prevention and control (IPC) in healthcare settings [5, 6]. Active carbapenemase-producing Enterobacterales (CPE) screening should be implemented on admission and periodically for high-risk patients to prevent the spread of carbapenem-resistant Enterobacterales (CRE) [7, 8].

Traditional culture methods are the most used for detecting CRE from rectal swabs [9]. These methods are cheaper and have high sensitivity and specificity, but they require 48–72 h laboratory turnaround time (TAT) [10, 11]. Such a long TAT increases unnecessary isolation and costly IPC measures in hospitals where preemptive isolation is performed and delays appropriate antimicrobial stewardship. PCR-based screening methods that target the main carbapenemase-genes (KPC, VIM, IMP, NDM and OXA-48) are gaining popularity due to their rapid TAT (a few hours), higher sensitivity if compared to culture, and the ability to detect non-culturable strains, making them a valuable tool for a prompt IPC, even though at a higher cost [11–13]. In clinical contexts where controlling transmission networks and outbreaks relies on the prompt identification and isolation of colonized patients, combining molecular screening with culture methods can enhance detection accuracy [14], providing a complete overview of local epidemiology to guide IPC interventions.

As no individual laboratory protocol is free of downfalls, in the absence of high quality, long-term evidence of the most cost-effective approach, the optimal laboratory workflow for active screening remains a matter of debate. With this study, we investigated the diagnostic performance and IPC implications of a simultaneous molecular and cultural screening approach. We aimed to highlight the real-world impact of a decision process based on rapid molecular surveillance on the in-hospital epidemiology and acquisition rates of CROs, incident invasive infections and associated costs, with particular focus on the possibility of sparing bedding resources by rapid exclusion of CRO colonization.

## Materials and methods

### Study design and population

This was a retrospective, single-center study conducted at Humanitas Research Hospital (ICH) (Rozzano, Milan). Data from routine CRO screening in adult patients (> 18 year) performed between 01/01/2017 and 31/12/2023 were collected, along with demographic, laboratory and clinical data, from electronic hospital records. All patients tested during the study period were eligible for inclusion in this study.

### Routine CRO screening

CRO screening is performed in patients at highest risk for invasive disease after colonization and at higher risk of transmission to other vulnerable patients. In particular: patients with a history of infection or colonization by CRE within the 12 months prior to the current hospitalization; patients transferred from other healthcare facilities (including acute care hospitals, intensive rehabilitation units, or long-term care facilities for the elderly); patients with a history of hospitalization within the previous 3 months; patients admitted for the first time to high-risk wards, such as Intensive Care Units (ICU), Oncology, Hematology, or Intensive Rehabilitation; patients undergoing dialysis or antineoplastic chemotherapy in the previous 12 months, or otherwise immunocompromised; patients carrying biliary stents and scheduled for surgery; and individuals identified as contacts of patients with CRE infection or colonization Screening is performed weekly for patients hospitalized in ICU and onco-hematology unit (EMA), *on demand* for patients admitted to Emergency Department (ED) or in any hospital ward upon Infectious Diseases (ID) specialist evaluation. Pre-emptive isolation is applied pending PCR results only for admittance to ICU or onco-hematological units. Upon positive PCR results, colonized patients are isolated in single room, under contact precautions.

Molecular testing by PCR is carried out using the Cepheid Xpert™ Carba-R on-demand assay, targeting KPC, VIM, NDM, OXA-48, IMP genes. Cultural analysis uses Brilliance™ CRE Agar, followed by identification of suspected colonies by MALDI-ToF and phenotypic susceptibility testing by M50 Automated Microbiology System NMIC-402-474 (Becton Dickinson Diagnostic Systems, Sparks, United States), interpreted according to the European Committee on Antimicrobial Susceptibility Testing (EUCAST) breakpoints (Breakpoint tables for interpretation of MICs and zone diameters. http://www.eucast.org). Both assays are performed starting from the same rectal swab. Decision of isolation precautions are taken based on the molecular result. Discordant cases are managed individually, upon consultancy within the Hospital Infection Committee.

### Definitions of infection events

Bloodstream infections (BSIs) were defined upon positivity of blood cultures (BC) performed in accordance with routine ICH procedures and guidelines [5], after exclusion of contamination events. Contaminations were defined by the positivity of only one BC bottle or 2 BC bottles from the same set for a common contaminant organism: coagulase-negative Staphylococci (CoNS), Bacillus spp., Corynebacterium spp., Propionibacterium spp., Micrococcus spp., viridans streptococci (*S. mitis*,* S. sanguinis*,* S. mutans*,* S. anginosus*,* S. constellatus*,* S. intermedius*), *Aerococcus* spp.

Infection events were included in the analysis whether diagnosed after surveillance screening, in the context of the same hospitalization. Multiple events by the same pathogen within 14-days were considered as a single event.

### Statistical analysis

For descriptive analysis, categorical variables were shown as absolute count and quantitative variables as median. The variables were compared using Fisher’s exact test or Chi-squared test. Turnaround times were compared using Wilcoxon signed-rank test due to the non-normal distribution of data. The Confidence Interval (CI) had been set to 95%, and a probability (p) value of 0.05 was considered statistically significant.

PCR was considered positive if one or more targeted resistance markers were detected, and negative otherwise. The diagnostic performance analysis (sensitivity, specificity, predictive values, likelihood ratios, Cohen’s Kappa) was conducted using corresponding culture results as reference. Temporal trends in per-patient colonization rates and relative prevalence were assessed using the non-parametric Mann-Kendall trend test.

Cox survival analysis was used to estimate the risk of incident BSIs during the same hospitalization.

Data were analyzed with R-statistical software (v. 4.3.2, R Core Team (2024), embedded in R Studio v.3.7.5, and STATA 18 software (Stata Statistical Software: Release 18. College Station, TX: StataCorp LLC).

### Cost analysis

The process evaluation encompasses both direct and opportunity costs for each approach, including personnel costs associated with swab processing, materials and reagents required for swab analysis, and the potential reallocation of resources made available through differences in result turnaround time between the different approaches.

The Personnel Cost involved in the tests processing includes both technicians and biologists. Task timing was measured using Activity-Based Costing methodology during a representative workday. A single day of observation was deemed sufficient due to the highly standardized and repetitive nature of the procedures. A detailed table of personnel cost is available in **Supplementary Table **S1.

Materials and Reagents Costs were determined through direct observation of technician and biologist usage patterns, with pricing subsequently provided by the supply chain department. A detailed table of material and reagents costs is available in **Supplementary Table S2**.

When calculating opportunity costs, for freed-up beds an occupancy rate of 85% and an average cost/bed of 250€ have been considered.

All costs were obtained from Human Resources and Purchasing offices.

## Results

### Positivity rates and diagnostic performance of Xpert™ Carba-R PCR

**Fig. 1:**
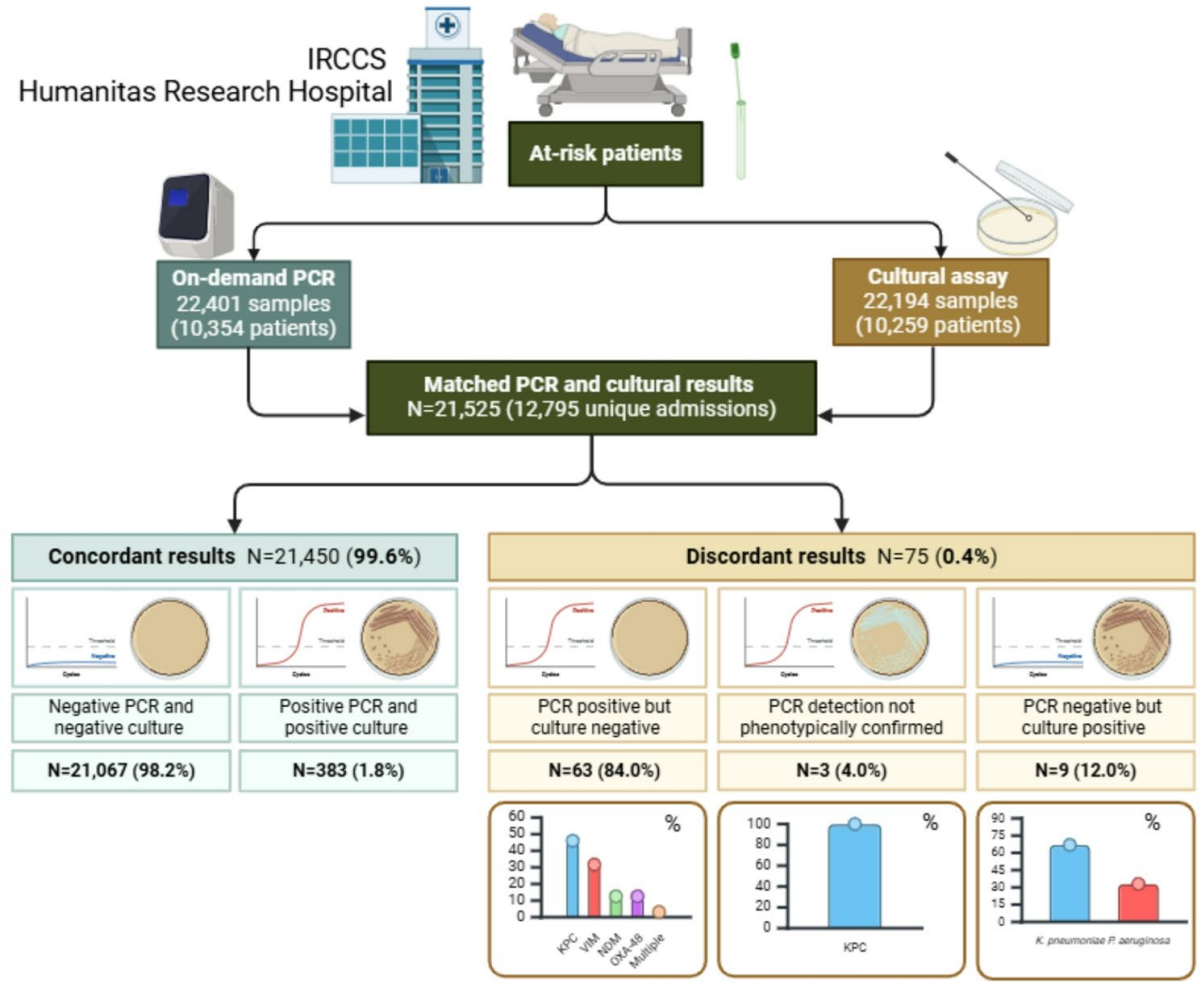
Study enrolment and agreement between PCR and cultural assay. *Created in BioRender. Lizier*,* M. (2025)* https://BioRender.com/bcc8dt8

Over 21,525 samples analyzed, overall agreement between PCR and culture was 99.6% (21,450/21,525) with a Cohen’s Kappa of 0.909 (95% CI: 0.89–0.93) (see Fig. 1 for enrollment and assay concordance). Negative PCR results were available in a median of 2.6 h versus 50.0 h by culture (*p* < 0.001), and resistance identification in 3.0 h versus 73.8 h for final report on antimicrobial susceptibility by cultural assay (*p* < 0.001).

PCR showed 99.7% specificity (95% CI: 99.61–99.76), a per-sample sensitivity of 97.7% (95% CI: 96.2–99.18), and a negative likelihood ratio of 0.02 for detecting targeted CRO colonization. Most discordances (66/75; 88.0%) were due to increased PCR detections in culture-negative samples. Notably, 32.1% of VIM-positive samples at PCR lacked corresponding microbial isolation. Only 9 samples (from 9 patients) were PCR-negative but culture-positive (7 *K. pneumoniae* and 2 *P. aeruginosa*). Even though a non-enzymatic resistance mechanism could be suspected in these cases, 100% of samples re-tested (*n* = 4) resulted in culture-negative, confirming initial PCR results. None of the 9 patients developed any CRO-related infection during hospital stay.

### Seven-years epidemiology of colonizing carbapenem-resistant organisms

After accounting for repeated testing (same patient, same hospitalization, same result), we analyzed 12,795 unique patient records. The median (IQR) age was 64.5 years (53.1–73.6), with 10.1% (*n* = 1,292) of the population older than 80 years. The 92.8% (*n* = 11,868) were Italian.

**Fig. 2:**
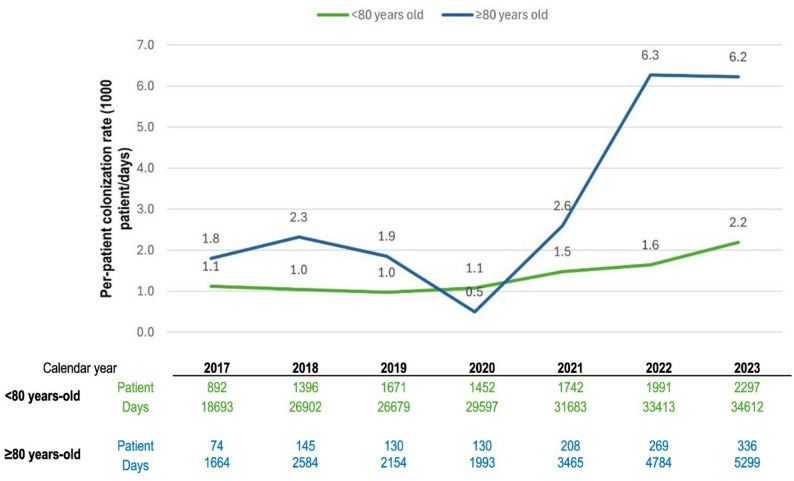
Per-patient colonization rate by carbapenem-resistant organisms, 2017–2023. The rate was normalized on 1000 days of in-hospital stay. Repeated tests (same patient, same hospitalization, same result) were excluded from the analysis. Rates are reported separately for patient younger (green) or older (blue) than 80 years

The per-patient CRO colonization rate was 1.50 per 1000 patient-days, with an increasing trend between 2019 and 2023 (*p* = 0.03), driven by patients aged ≥ 80 years, in which the rate rose, during the study period, from 1.1 to 6.2 per 1000 patient-days (Fig. 2). At logistic regression analysis, age ≥ 80 years was an independent predictor of CRO-colonization (OR 2.6; 95% CI: 2.04–3.36; *p* < 0.001) after correction for gender and year of testing (**Supplementary Table S3**).

**Fig. 3:**
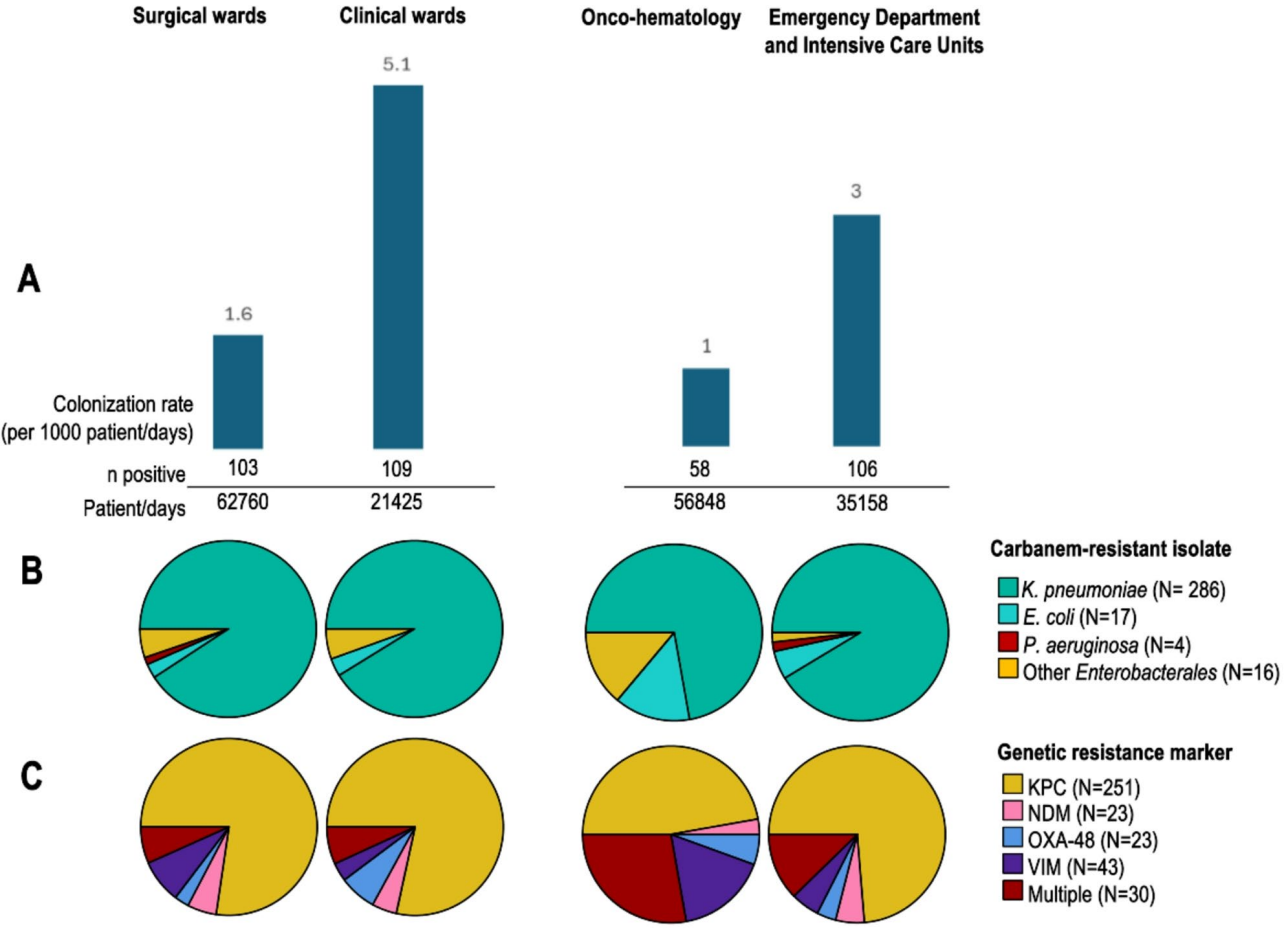
Epidemiology of carbapenem resistance by active screening, 2017–2023. **Panel A** reports colonization rates per 1000 patient/days in the clinical settings analyzed. Epidemiology of carbapenem-resistance isolates and genetic markers associated with resistance to carbapenems are reported in **panels B** and **C**, respectively. Only unique isolations for each microorganism or resistance gene were analyzed. Multiple resistance markers included: NDM-VIM, NDM-KPC, NDM-OXA48, OXA48-KPC, VIM-KPC, NDM-OXA48-KPC, and NDM-VIM-KPC

Colonization rates were higher in clinical wards (5.1 per 1000 patient/days) compared to surgical wards (1.6 per 1000 patient/days), Onco-hematology Unit (1.0 per 1000 patient/days) and Intensive Care/Emergency Departments (3.0 per 1000 patient/days) (Fig. 3, **panel A**). *K. pneumoniae* predominated (Fig. 3, **panel B**), accounting for 88% of isolated CROs, with no significant modification over the years (*p* = 0.87 for relative prevalence) (**Supplementary figure ****S1**). Resistance markers distribution reflected this epidemiology, with bla_KPC_ being the most detected, followed by bla_VIM_ gene (Fig. 3, **panel C**). Onco-hematology ward showed lower *K. pneumoniae* recovery (72.5% vs. 94.6% in the other wards, *p* < 0.001) and higher *E. coli* prevalence (14% vs. 3.8%, *p* = 0.003). Of 334 unique patients, 8.6% had isolates with multiple resistance genes, with *E. coli* and *K. pneumoniae* at 11.7% and 8.0%, respectively.

In the 7 years of the study, the rate of detection of PCR non-target pathogens was 0.04 (n/*N* = 9/21,525 samples), represented by carbapenemase producing *Acinetobacter baumanii* (*n* = 9).

In high-risk wards with systematic screening, incidence of CRO acquisition rates were low: 1.3 per 1000 patient-days in ICUs and 0.32 in Onco-hematology (median stay 11 and 7 days, respectively) (Table 1). Over the analysis period, the rate of CRO acquisition in Onco-hematology showed a progressively decreasing trend from 1.03% in 2017 to 0.67% in 2023 (*p* = 0.07) (**Supplementary figure S2**). For ICU, the analysis of CRO acquisition rate trend was not possible due to the low number of isolates tested in the first years.

**Table 1:** Incidence of CRO in ICU and ONC-EMA, 2017–2023

Characteristics	Intensive Care Unit	Onco-hematology Unit
CRO carriage rate on admission, % (n/N)	0.94 (67/7,065)	1.16 (41/3,526)
Acquisition rate of CRO^a^, % (n/N)	2.29 (33/1,438)	0.7 (17/2,406)
Total duration ^a^, person/days	25,329	52,916
Incidence of CRO acquisition ^a^, person/days	1.3/1000	0.32/1000
Median length of stay since admission to the 1st CRO detection (IQR)^a^, days	11(16.5)	7 (0.0)

^a^ Calculated-on patients with ≥ 6 days of in-ward stay

### Incident bloodstream infections in colonized and non-colonized patients

During the study period, a total of 1,181 unique BSIs events were diagnosed by positive blood-cultures (BCs) in the analyzed population, including 12 (1.0%) identified at admission to ED. *Enterobacterales*, *A. baumannii* and *P. aeruginosa* accounted for 42.5% of total BSIs cases (*n* = 503), and in the 14.9% of these (*n* = 75) were phenotypically resistant to carbapenems.

Outside the ED, the incidence of at least one BSI by CRO was 0.29 per 1000 patient/days, a median of 12 (IQR: 7-24) days after the hospital’s admission.

In high-risk wards with systematical screening, most BSIs by CRO occurred in patients with no identified colonization (76.9% ICU, 52.6% EMA), caused mainly by *A. baumannii* (60% ICU, 40% EMA). Yet, a multivariable Cox regression model showed that CRO colonization acquisition during the hospitalization and pre-existing bla_NDM_ colonization were independent predictors of CRO-BSI in ICU and onco-hematology (aHR = 8.11, 95%CI = 2.38–27.61, and aHR = 6.23, 95%CI = 1.05–36.99, respectively), after adjusting for age, sex, year of enrollment, time of CRO colonization acquisition, and type of carbapenemases (Table 2).

**Table 2:** Univariable and Multivariable Cox regression model for predictors of CRO-BSI during hospitalization in ICU and Onco-Hematological setting

	Univariable Analysis				Multivariable Analysis	
	HR	95%CI	*p* value	aHR	95%CI	*p* value
Age	1.01	0.99–1.04	0.181	1.02	0.99–1.04	0.128
*Year*						
2017	1					
2018	1.11	0.24–5.00	0.89			
2019	0.33	0.03–3.25	0.347			
2020	1.51	0.37–6.04	0.56			
2021	1.54	0.38–6.21	0.539			
2022	1.52	0.38–6.12	0.55			
2023	3.92	1.10–13.8	0.038			
CRO - Colonization acquisition	18.32	9.00–37.27	< 0.001	8.11	2.38–27.61	**< 0.001**
*Type of carbapenemase*						
VIM	6.81	1.61–28.7	0.009	0.69	0.09–5.27	0.723
KPC	13.67	6.62–28.2	< 0.001	2.95	0.86–10.14	0.084
NDM	25.3	7.65–83.7	< 0.001	6.23	1.05–36.99	**0.044**
OXA-48	13.4	1.82–99.3	0.011	0.79	0.04–13.83	0.872
Multiple	37.1	11.2–122.3	< 0.001	4.15	0.47–36.6	0.2

Legend: HR, Hazard Ratio; CI, Confidence Interval; aHR, adjusted Hazard Ratio; CRO, carbapenem-resistant organism. In bold statistically significant p-values

The colonization to BSI ratio showed no significant trend over the seven years of observation.

### Cost analysis for transition from a culture-based to a molecular based screening

When considering direct costs, the molecular approach incurs substantially higher direct costs than the cultural approach (€8.40 representing a 32% increase for negative outcomes, and €9.80 representing an 11% increase for positive outcomes). While the molecular approach utilizes an expensive testing kit compared to the simple medium required for cultural determination, it does reduce health professional time requirements. Considering the cost per single determination, using only the molecular test increases direct cost by 31% in comparison to using the cultural approach given the same clinical outcome. The cost opportunity analysis highlights a mean time advantage of approximately 45.2 h per sample (± 12.3 h standard deviation) for the molecular approach. A paired t-test confirmed the statistical significance of the molecular technique’s time advantage (t = 52.47, *p* < 0.0001), with an average time saving of approximately 42.5 h per sample. The quicker turnaround times of the molecular test, when the result is negative, allows to free up days of stay that can be reallocated to treat additional patients. In the study period, considering the total days of stay in ordinary wards, an average unit cost per bed-day of €250, and an average bed occupancy rate of 85%, the adoption of only molecular tests could potentially have freed up resources worth a total of €4 million, which translates to the accommodation of approximately 3,134 additional patients. (Table 3).

**Table 3:** Cost analysis for transition from a culture-based to a molecular-based plus cultural confirmation screening approach

Year	Tests with negative outcome	Tests with positive outcome	Total tests	Total Cost of cultural approach (€)	Total cost of molecular approach (€)	Delta cost (%)	Delta time (*n* days)	No. beds involved (pre admission set)	Days of stay intensive care units	Days of stay ordinary wards units	Number of beds freed-up	Total value of freed up resources in €*	Actual bed days used ordinary ward units	Additional patients treated
2017	1.661	22	1.683	45.873	60.039	31%	2.517,5	104	1.062	1.352	4	287.247	1.149,0	185,3
2018	2.461	34	2.495	68.087	89.091	31%	4.668,4	135	1.659	2.875	8	610.844	2.443,4	394,1
2019	2.95	24	2.974	80.181	105.194	31%	5.391,4	195	1.58	3.616	10	768.433	3.073,7	495,8
2020	2.958	31	2.989	80.992	106.141	31%	6.121,8	185	2.585	3.352	9	712.241	2.849,0	459,5
2021	3.431	66	3.497	96.516	125.98	31%	5.914,4	154	2.363	3.398	9	722.093	2.888,4	465,9
2022	3.732	78	3.81	105.515	137.625	30%	6.935,6	158	2.686	4.092	11	869.446	3.477,8	560,9
2023	3.936	141	4.077	116.313	150.753	30%	7.325,2	187	2.961	4.177	11	887.506	3.550,0	572,6

* Only days of stay ordinary wards have been accounted, with a bed-cost/day of 250 € and an average bed occupation of 85%

## Discussion

Seven years of hospital-wide active surveillance demonstrated that analytical performance of rapid molecular swab testing is similar to culture-based, with the added advantage of providing clinical responses much more quickly. On the long term our screening approach translated in a very low rate of in-hospital CRO spread that was identified as the major risk factor for BSI development in high-risk patient, and saved bed resources, enabling the hospital to treat additional patients.

From an IPC perspective, rapid CRO screening is essential for timely patient care [13]. Our workflow provided negative PCR results in a median of 2.6 h—compared to 50 h for culture (*p* < 0.001) and 4–7 h for other commercial PCR assays [14]. Since many hospitals isolate patients pending CRO results, rapid screening would significantly reduce unnecessary isolation or limited it to just a few hours for negatives. In our setting, the use of Xpert Carba-R PCR eliminated pre-emptive isolation in clinical wards and the ED, saving about 10 bed-days per year and enabling treatment of additional patients with the same resources. In high-risk wards such ICU and ONC-EMA, rapid systematic surveillance and prompt isolation of positive patients kept CRO colonization incidence at low levels, with a decreasing trend in the rate of CRO acquisition highlighted in ONC-EMA from 1.03% in 2017 to 0.67% in 2023 (*p* = 0.07). In such setting, CRO acquisition was found to be the main risk factor for the development of a CRO-BSI during the same hospitalization (aHR [95% CI]: 8.11 [2.38–27.61]; *p* < 0.001) (**Supplementary Table S3**). In this light, maximizing the effectiveness (and speed) of IPC measures aimed at preventing in-hospital colonization represents the intervention with the greatest impact on the risk of developing invasive infections, preserving fragile and highly-treated patients with poor therapeutic chances in which incident infections can cause an increase in length of stay in intensive areas at higher costs, with potentially poor outcomes.

In the 7 years of the study, the rate of detection of non-target CRO pathogens by culture was 0.04 (n/*N* = 9/21,525 samples, all positive for *Acinetobacter baumannii*), supporting the representativeness of the PCR panel used for our epidemiological context. In line with previous studies [14, 16] and general epidemiology [24], whole genome sequencing of two of our *A. baumannii* strains revealed the presence of OXA-23 as carbapenem resistance marker, as elsewhere described [17].

In our hospital setting, the overall CRO-carriage rate was of 2.07%, consistent with other experiences, and slightly below the expected 3–7% Italian prevalence [8, 14, 16]. The increase in CRO detection observed during 2023, especially in patients > 80 years-old (Fig. 2), is in line with estimates of our geographical area [14], and proceeds in step with the increase in the complexity of patients treated in our institution over the last few years. Higher colonization rates in older patients underscore the need to identify setting-specific CRO risk factors and implement targeted screening, optimizing IPC resources beyond traditional populations.

Even though *de novo* CRO-colonization acquisition is the primary risk factor for subsequent BSI development, pre-existing bla_NDM_ colonization increases the risk of BSI from the same pathogen by 6.23 times (CI 95%: 1.05–36.99), a risk not observed with colonization by other CRO. NDM-carbapenemases were identified as a main driver of BSIs also in another large retrospective study conducted in Italy, probably due to the specific virulent clone circulating in that region [19]. Unfortunately, the lack of WGS data on our NDM-carrying clones didn’t allow for their genomic characterization and confirmation of increased virulence.

Epidemiological assessment is a critical aspect of IPC, as CROs heterogeneity extends beyond prevalence [21]. Our CRO epidemiology did not significantly changed over the last 7 years, with the 88% of detected CRO being *K. pneumoniae*, in accordance with its high prevalence in Italy [16, 22], and the most detected gene being bla_KPC_ (73.9%) [14]. The peak in bla_VIM_ prevalence during year 2020 (37.5% vs. <15% in 2021–2023) was related to a possible outbreak in ICU, rather than an epidemiological shift. Indeed, even though no WGS data was available, 7 out of the 12 bla_VIM_-positive patients of 2020 were detected in ICU in a two-weeks period. Notably, 32.1% of bla_VIM_ cases overall were culture-negative, including 2 patients involved in the possible 2020 ICU outbreak. A suboptimal categorical agreement (60%) between PCR and culture in bla_VIM_ detection has been recently reported in a similar setting, by using a different PCR kit [14]. This suggests that high-risk settings with expected enriched circulation of bla_VIM_ can be advantaged using PCR for surveillance, rather than culture alone.

Compared to other Italian settings and the EUSCAPE study, we underline the poor recovery of bla_NDM_ isolates and the extremely rare (if any) circulation of bacteria using other mechanisms of resistance, including bla_IMP_ or non-enzymatic [14, 21, 23, 24]. The increase in the number of NDM and NDM/OXA-48 *K. pneumoniae* we observed in 2023 has been previously notice also by another study from Northern Italy, apparently not related to any possible outbreak [24]. As mentioned previously, molecular typing data for the NDM-positive isolates in this study are unavailable, precluding further analysis.

The economic analysis revealed an increase in direct costs associated with the use of molecular testing, due to higher kit costs. However, the quicker turnaround time of the PCR technique, allow for the earlier discontinuation of isolation with negative results, freeing up bed-days that can be reallocated to treat additional patients. Initially, the protocol required patients with suspected infection to be isolated in single rooms from the moment of swab collection until results became available. With PCR testing, patients can be transferred to general wards 1–2 days earlier, optimizing the use of hospital beds, a critical and limited resource. By freeing up beds without compromising clinical outcomes, the molecular approach creates significant value. However, the freed bed-days following negative results can be reallocated primarily in ordinary wards, indeed in settings such as intensive care units (ICUs), these time savings cannot typically be reused for other patients, as ICU patients generally require ongoing care regardless of the swab result. In such cases, the advantage of molecular testing lies in its clinical value: providing rapid, highly sensitive results that support continuous monitoring and control of CRO transmission, ultimately reducing the risk of in-hospital bloodstream infection (BSI) development. Indeed, while appropriate initial empirical therapy is associated with lower in-hospital BSI mortality [25], patients infected with resistant pathogens are more likely to receive inadequate treatment [25, 26]. In this context, and in line with MSSA/MRSA surveillance [27], rapid CRE screening could be a valuable tool for guiding empirical therapy, particularly in suspected BSI cases under infectious disease supervision, as suggested by a few studies [28, 29]. To sustain this hypothesis, further in-depth and wider analyses are foreseen. On the other hand, given the incremental costs, the absence of recovered resources, and the lack of urgency for rapid results, the use of molecular methods in in settings like pre-admission appears unnecessary. Importantly, we acknowledge that the economic advantages identified primarily represent a theoretical increase in hospital capacity rather than immediate financial savings. Furthermore, given the increase in direct costs, additional studies are necessary to evaluate the financial feasibility of molecular testing in resource-limited healthcare settings.

This study has certain limitations. The retrospective nature of the study and the size of the population involved precluded the retrieval of detailed clinical data, particularly regarding patients’ clinical severity and underlying comorbidities, beyond the indirect information inferred from the hospitalization unit (i.e., EMA or ICU). As a result, these variables could not be incorporated into the risk analysis for incident BSIs, nor could they be leveraged for a more in-depth epidemiological characterization of colonization patterns. The monocentric design and the limited incidence of BSIs during hospitalization contributed to restrict our ability to fully assess the impact of a rapid screening approach on the prevention and management of hospital-acquired infections, along with the absence of baseline BSI incidence data prior to the implementation of active surveillance in 2017. The analysis on colonization acquisition and incident infections was confined to EMA and ICU units due to the heterogeneity of screening strategies (systematic vs. on-demand) across different hospital wards, which introduced an excessive risk of bias in medical and surgical departments. Lastly, a minor but noteworthy limitation is the restricted gene panel of the Cepheid Xpert™ Carba-R assay, which, although of limited impact in this study, does not fully capture the actual molecular epidemiology (e.g., ESBL) and may limit the detection of less frequent resistance mechanisms, such as OXA-23.

Despite these limitations, this seven-year study underscores the value of implementing rapid molecular screening for CRO colonization. The data generated through this active surveillance serve as a crucial resource for understanding the local epidemiology of CRO, including the dynamics and dissemination of carbapenemase genes, while also providing insights into the cost-effectiveness and clinical impact of such surveillance strategies on hospital-acquired BSIs. These findings offer a foundation for healthcare policymakers to support the implementation and optimization of molecular surveillance on a hospital-wide scale and pave the way for future studies focusing on screening-guided antimicrobial stewardship approaches.

## Supplementary Information

Below is the link to the electronic supplementary material.

Supplementary Material 1

## Data Availability

The datasets used and/or analysed during the current study are available from the corresponding author on reasonable request.
