# Supplementary material for: Clinical and cost benefit of rapid molecular screening for carbapenem-resistant organisms: a seven-year analysis in a tertiary hospital
Source: Antimicrob Resist Infect Control. 2026 May 10;15:101. doi: 10.1186/s13756-026-01750-7 (PMC13464901; doi:10.1186/s13756-026-01750-7)
Supplement: Supplementary file 1 [file 13756_2026_1750_MOESM1_ESM.docx]

| **Supplementary table S1. Personnel cost summary** | | |  |  |  |  |
| --- | --- | --- | --- | --- | --- | --- |
|  |  |  |  |  |  |  |
| **Personnel cost** | **Lab technician** | | **Biologist** | | **Total** | |
|  | Time/test (h) | Cost/test (€) | Time/test (h) | Cost/test (€) | Time/test (h) | Cost/test (€) |
| Cultural test – negative | 0.8 | 17.7 | 0.2 | 7.4 | 1 | 25.1 |
| Cultural test – postiive | 1.8 | 39.8 | 1.2 | 44.5 | 3 | 84.3 |
| Molecular test – negative | 0.2 | 4.4 | 0.2 | 7.4 | 0.4 | 11.8 |
| Molecular test - positive | 1.2 | 26.5 | 1.2 | 44.5 | 2.4 | 72 |

| **Supplementary table S2. Reagent cost summary** | | |  |
| --- | --- | --- | --- |
|  |  |  |  |
| **Cost of materials and reagents** | **Cost/test (€)** |  |  |
| Cultural | 1.4 |  |  |
| Molecular – negative | 23.1 |  |  |
| Molecular – positive* | 24 |  |  |
|  |  |  |  |
| *positive molecular tests require cultural test for pathogen identification | | | |

**Supplementary Table S3.**

| **Variables** | | **Prevalence, %** | **Univariate logististic regression** | | | **Multivariate logististic regression** | | |
| --- | --- | --- | --- | --- | --- | --- | --- | --- |
|  |  |  | **Odds-ratio** | **95% confidence interval** | **P-value** | **Odds-ratio** | **95% confidence interval** | **P-value** |
| Male sex | | 59.2 | 0.791 | 0.637-0.981 | 0.03 | 0.794 | 0.639-0.986 | 0.04 |
| Age ≥80 years | | 10.1 | 2.771 | 2.162-3.552 | <0.001 | 2.618 | 2.040-3.360 | <0.001 |
| Year of testing | *2017* | 7.6 | Reference | | | Reference | | |
|  | *2018* | 12.1 | 0.882 | 0.520-1.496 | 0.64 | 0.866 | 0.510-1.471 | 0.59 |
|  | *2019* | 14.1 | 0.663 | 0.386-1.141 | 0.14 | 0.667 | 0.388-1.149 | 0.14 |
|  | *2020* | 12.4 | 0.835 | 0.491-1.422 | 0.5 | 0.826 | 0.485-1.408 | 0.48 |
|  | *2021* | 15.3 | 1.174 | 0.724-1.903 | 0.52 | 1.115 | 0.686-1.812 | 0.66 |
|  | *2022* | 17.8 | 1.523 | 0.962-2.411 | 0.07 | 1.441 | 0.909-2.285 | 0,12 |
|  | *2023* | 20.6 | 1.738 | 1.111-2.718 | 0.02 | 1.613 | 1.030-2.528 | 0.037 |

**Supplementary figure S1. Prevalence trend of carbapenem resistance isolates, 2017-2023**.

Prevalence of unique positive isolates by species, over all unique positive isolates. Other Enterobacterales include *Citrobacter freundii*, *Citrobacter koseri*, *Enterobacter cloacae*, *Klebsiella aerogenes*, *Klebsiella oxytoca*, and *Klebsiella variicola*.

**Supplementary figure S2. Trend of CRO-acqusition rate in Onco-haematology, 2017-2023.**

^a)^ Only patients negative at admission, and which at least 6 days of in-ONC-EMA stay have been included.
